# Scientific writing and editorial policies and procedures of the Journal of Veterinary Internal Medicine

**DOI:** 10.1111/jvim.16987

**Published:** 2024-01-09

**Authors:** Stephen P. DiBartola, Kenneth W. Hinchcliff

## 1 GENERAL COMMENTS ON SCIENTIFIC WRITING

Clinicians and scientists are busy people with many demands on their time. The material they choose to read should clearly convey the specific information they need in a way they can easily understand.

Good scientific writing, which differs from creative writing, is a skill that can be developed and improved with practice. To communicate effectively and without ambiguity, good scientific writing must be clear, precise, concise, and follow a logical progression of thought. It should use plain language and avoid jargon. Precision, saying exactly what one means to say, is critical in preventing ambiguity. Information should be relevant and presented in a way that can be easily understood and assimilated by the reader. The reader should not have to struggle to understand the writer's meaning.

Scientific writing should be concise. The costs of printing and distributing a paper journal have been eliminated in today's world of electronic publishing, and thus some authors believe word limits are no longer important. We do not agree. We believe that busy clinicians and researchers budget the time they spend reading the veterinary literature, and thus word limits remain important. It is incumbent on authors to communicate the relevant information not only completely but concisely.

Writing style should be appropriate for the nature of the work and its intended audience. Writers of fiction sometimes strive to mimic the nonlinear thought processes of humans with results that challenge their readers, but writing a scientific report is fundamentally different from writing a novel. The writer of a scientific paper should strive to make its content easily understandable to the reader. As theoretical physicist Paul Dirac reportedly said: “In science one tries to tell people, in such a way as to be understood by everyone, something that no one ever knew before. But in poetry, it's the exact opposite.”

In scientific writing, one should favor the active voice and use short declarative sentences. However, varying sentence length can improve readability and help maintain the reader's attention. Long paragraphs should be avoided. As E.J. Huth (longtime editor of the Annals of Internal Medicine and founding member of the International Committee of Medical Journal Editors) said: “A paragraph should end when what it promised at or near its beginning has been delivered.”

Avoid run‐on sentences. A run‐on sentence consists of 2 or more independent thoughts connected improperly. Such sentences can be nearly impossible to understand because the reader cannot determine where one thought ends and the next one begins. In simple terms, a run‐on sentence can be fixed by placing periods at the end of each independent thought to create several shorter sentences. Avoid long introductory prepositional phrases that add nothing to the sentence. For example, one nearly always can strike the introductory phrase, “It should be emphasized that …” from the beginning of a sentence without any loss of meaning. Avoid expletive constructions that begin “There are …” or “This is …”. Such sentences can be revised by rearranging a few words in the sentence and supplying a more concrete subject. For example, “This is the most common lesion of histoplasmosis” can be changed to “Pyogranulomatous inflammation is the most common lesion of histoplasmosis.”

Good writing is the product of rewriting, so just get started. Sometimes concern about the final form of written expression paralyzes a writer—that is, the writer worries that what is written first will be what appears in the published article. Just write what first comes to mind and then go back and revise the text—over and over again. Authors should thrive on revision—editing their own work until it cannot be further improved.

## 2 STYLISTIC MATTERS RELATED TO THE JOURNAL

Always read the Journal's instructions to authors. Carefully consulting these instructions can minimize the number of changes you will be asked to make during revision and editing of your manuscript.

The Journal does not use the terms canine, feline, equine, and similar terms as nouns. The terms canid, felid, equid, and similar terms can be used as nouns, but typically include other members of the family in question (ie, canids include dogs and wolves; felids include lions, tigers, cheetahs, and other cats; and equids include horses, asses, and zebras). When used as adjectives, the words canine, feline, equine, and similar terms should only modify words that originate from the species in question. For example, feline plasma or equine heart would be permissible whereas equine asthma, canine epilepsy, or feline lymphoma, when referring to asthma in horses, epilepsy in dogs or lymphoma in cats, would not. Difficulties arise however when these adjectives are used to refer to diseases, because the diseases in question are not dogs, cats, or horses. The Journal does not permit such use, except in rare instances as determined by the editors.

Use the present tense to describe knowledge that is the result of previous publications (eg, “Cats with FIV infection are more likely to die prematurely.^4‐9^”) and the past tense to describe results of the current study (eg, “We determined that FIV infection causes stomatitis in cats”).

Do not use prefatory phrases when presenting results described in earlier reports. For example, “*it was previously shown that fat cats die at a younger age*” or “*it is well known that all dogs with arthritis are lame*.” These statements can be rephrased more simply as: “*fat cats die at a younger age*” or “*dogs with arthritis are lame*.” Ensure that the emphasis of the sentence is on the fact that you intend to convey, not that research was done. For example, rather than “*research shows that dogs cannot see blue*,” write “*dogs cannot see blue*.”

Avoid constructs such as “*Smith et al* ^*23*^ *showed in 1997 that dioxin caused lymphoma in dogs, which was supported by Jones et al in 1999* ^*24*^ *who showed that dioxin induced genetic mutations in dogs*” because they are cumbersome and focus attention on the author rather than the facts. A preferred grammatical construct is “*Dioxin causes lymphoma in dogs, possibly by induction of genetic mutations*.^*23,24*^” because this wording presents only the facts being conveyed, is briefer, and does not distract the reader. Attribution is maintained by appropriate use of citations.

In the Journal, all numbers are expressed as numerals, including numbers between 0 and 10. Thus “one” is spelled out only when used as a pronoun (eg, “One should always use numerals when expressing numbers in a manuscript to be submitted to the Journal of Veterinary Internal Medicine”).

Do not use personal pronouns (eg, he, she, him, her) to refer to animals. Instead, use “dog,” “cat,” “horse,” or “it.”

The Journal's convention is to omit leading zeros from *P* values and to express *P* values 2 places to the right of the decimal. Thus, “*P* = .02” and not “*P* = 0.0176.”

Dosage regimens should be indicated, for example, as “q8h” or “q12h” rather than TID or BID.

The Journal does not report “trends”—that is, data that approaches statistical significance but is not significant. If a result was not significantly different based on statistical analysis, it was not different.

The Journal does not publish tables with individual animal results in the body of the manuscript. Such information can be included in the supplemental information.

Use of nonstandard abbreviations should be minimal. Abbreviations should be spelled out at first use in the body of the manuscript followed by the designated abbreviation within parentheses. From that point on in the manuscript, only the abbreviation should be used for the term in question. However, abbreviations should not be used to start sentences. Authors should include all abbreviations used in the manuscript in the “list of abbreviations” at the beginning of the manuscript and be sure not to use the same abbreviation for 2 different terms in the manuscript (eg, do not use “bpm” to refer both to “beats per minute” and “breaths per minute”). Finally, the Journal does not spell out and rather uses only abbreviations for a selected number of terms, such as CBC, ECG, ELISA, IM, IV, PCR, PCV, PO, and SC.

Authors should use appropriate symbols as needed. Do not use a lower case “u” for “micro” but rather use the symbol for the Greek mu (μ). Do not use a lower case “x” for “multiply by” but rather use the appropriate symbol (×).

Finally, strive for modesty! Do not make claims of priority such as “our study is the first to …” or “ours is the first report of a case of …” because you can never be certain of such a statement, and the claim is neither essential nor sufficient to justify your study.

## 3 WORD CHOICE AND COMMONLY CONFUSED WORD PAIRS

### 3.1 “A total of”

It is preferable to spell out a number at the beginning of a sentence. However, if the number is large and unwieldy, it is permissible to use the expression “A total of …” to avoid spelling out a very large number.

### 3.2 Accuracy/Precision

“Accuracy” refers to the extent of correctness of a measurement; “precision” refers to the degree of refinement with which a measurement is made.

### 3.3 Adverse effect/Side effect

A “side” effect is an unintended effect—good, bad, or neutral. If you are referring to a bad side effect, use the adjective “adverse.”

### 3.4 Affect/Effect

“Affect” is almost always a verb (meaning “to influence”). “Effect” is usually a noun (meaning “result”). When used as a noun, “affect” refers to expression of emotion (eg, “he has a flat affect”). When used as a verb, “effect” means “to bring about or cause.”

### 3.5 After/Following

Use “after” rather than “following” when time sequence is involved; it is more precise. “Following” can be interpreted spatially, such as “the dog was following the cat down the street.”

### 3.6 Among/Between

Use “between” when comparing 2 things; use “among” when comparing more than 2 things.

### 3.7 Because of/Attributed to/Caused by/Due to

“Because of,” “caused by,” or “attributable to” are preferred to “due to.”

### 3.8 “and/or”

Do not use the expression “and/or” because it is ambiguous (ie, were both “A” and “B” required or only 1 of them?). Use “A or B,” “A and B,” or “A or B or both,” but not “and/or.”

### 3.9 Because/Since

Don't use “since” when you mean “because.” “Since” suggests a temporal relationship rather than a causal relationship.

### 3.10 Before/Prior to/Subsequent to

Don't use “prior to” and “subsequent to.” Rather, say “before” and “after.”

### 3.11 Biopsy/Biopsy sample (or specimen)

A “biopsy” is the procedure used to collect a sample, the “biopsy sample” or “biopsy specimen.”

### 3.12 Case/Patient

Do not use the word “case” to describe the animal itself. Use “dog,” “cat,” “horse,” or the appropriate name of the species. A “case” is a particular instance of a disease. Reserve the word “patient” for describing humans with disease, not animals.

### 3.13 Clinical sign/Symptom

A “symptom” is something perceived by a patient and related during the history given by the patient (ie, a “symptom” applies only to human patients who can express how they feel). Use the term “clinical sign” when referring to manifestations of disease in an animal.

### 3.14 Compared to/Compared with

The expression “compared to” is used to emphasize the similarity between 2 things (ie, likening them *to* each other) whereas “compared with” is used to emphasize that the differences between 2 things are more important than their similarities (ie, contrasting them *with* each other).

### 3.15 Concentration/Level

Do not use the term “level” when you mean “concentration.” For most analytes, “concentration” is the correct term, but for enzymes the expression “activity” is used. So, serum creatinine concentration but serum creatine kinase activity.

### 3.16 Depressed/Lethargic

Do not use the term “depressed” to refer to an animal's mental status because “depression” is subjective and refers to a perceived symptom. Instead, use “lethargic” because it is a clinical sign that can be objectively observed.

### 3.17 Dilation/Dilatation

Some sources indicate that these words mean the same thing and can be used interchangeably, but the Journal prefers “dilatation” when referring to pathologic stretching of a structure (eg, “dilatation of the left ventricle”). “Dilation” is preferred when referring to an active process by which a structure is enlarged (eg, “the stricture was relieved by balloon dilation of the esophagus”).

### 3.18 Dose/Dosage

A “dose” is the total amount of a drug given a one time (eg, 100 mg or 10 mg/kg) whereas “dosage” refers to the regimen of repeated administration (eg, 10 mg/kg q12h for 14 days).

### 3.19 Duration/Length

“Length” refers to physical size (eg, the “length” of the small intestine) whereas duration refers to measurement of a time period (eg, the “duration” of hospitalization).

### 3.20 Employ/Utilize/Use

“Use” is shorter than “utilize” and means the same thing. “Use” is preferable to “employ” for the same reason.

### 3.21 Ensure/Insure

“Insure” means to provide insurance for; “ensure” means to make some future event certain. In most instances in scientific writing, “ensure” is the preferred term.

### 3.22 Farther/Further

“Farther” suggests distance; “further” means “something additional.”

### 3.23 Feces/Stool

“Feces” refers to waste matter discharged from the large intestine; a “stool” is a seat without back or arms. You may step on the latter, but you would prefer not to step on the former!

### 3.24 Fewer/Less

Use “fewer” if the things you are describing are discrete units; use “less” when what you are describing does not consist of discrete units. For example, “fewer” dogs but “less” anxiety.”

### 3.25 Frequency/Incidence/Prevalence

“Incidence” is the percentage of individuals in a population that develop a condition during a specified period of time (ie, the number of new cases of disease that develop during the time period.) “Prevalence” is the percentage of individuals in a population or sample group that have the condition at a given point in time. When referring to the number of animals manifesting a particular finding in a small group of individuals it is preferable to use the “frequency.”

### 3.26 Gender/Sex

Do not use the word “gender” to refer to the biological sex of the animal—use the word “sex” instead. Gender is socially constructed term applied to the behavior of humans.

### 3.27 Impact/Effect

Avoid use of the word impact, unless it refers to the physical event of striking (eg, “the meteor impact caused extinction of the dinosaurs”). If you are writing about the effect of an action or event on a variable, rather than writing “doxorubicin impacted myocardial function,” be specific and write “doxorubicin decreased myocardial contractility”. Also, do not assume the word “impact” will be taken to mean a negative effect or that readers will understand the nature of the “impact.” Be specific and precise about the effect (“impact”) of the action or event.

### 3.28 Increased/High

Use of “increased” implies a change over time. When reporting a result that is “increased,” the referent should be clearly stated (eg, Day 3 results were increased over Day 1 results). When describing the results of a single time point, use “results were above the reference range” or “results were higher in affected animals” rather than “results were increased.” The same is true for “decreased” and “lower than.”

### 3.29 Mortality rate/Case fatality rate

Mortality rate refers to the death rate in a population regardless of cause (the crude mortality rate) or caused by a specified disease (specific mortality rate). Case fatality rate refers to the proportion of animals with a specific disease that die during a specified time frame. For example, parvoviral enteritis might kill 1 dog in a population of 10 000 dogs (the mortality rate) but 1 dog in 10 that develop the disease within a specified time frame (the case fatality rate).

### 3.30 Mortality/Death

Do not confuse mortality and death. The death of an individual is the end of that individual's life and not the individual's “mortality.” Mortality refers to the state of being mortal (ie, a state of being in which one can die). “Mortality” and death are not synonymous. Do not use euphemisms for the end of life (eg, “passed away”). Be clear and use “died” or “was euthanized,” as appropriate for the context.

### 3.31 Nouns versus Adjectives

The words estrus, mucus, phosphorus, and villus are *nouns*; the words estrous, mucous, phosphorous, and villous are *adjectives*. Thus, “mucus” in the trachea but “mucous membranes,” and a “villus” in the intestine but “villous” atrophy.

### 3.32 Parameter/Variable

A “parameter” is one of a set of measurable factors (often numerical) that characterize a system and define its behavior (eg, heart rate and blood pressure are “parameters” of cardiovascular function) whereas the term “variable” refers to a member of a group of measurements that are not necessarily related. When in doubt, use “variables.”

### 3.33 Pathology/Lesion

Pathology refers to study of the disease. Diseases produce lesions, not pathology. In this context, instead of “pathology,” use terms such as “disease,” “lesion,” “abnormal function,” or “abnormality.”

### 3.34 Population/Sample/Cohort/Group

Investigators study a “sample” from a “population” of animals. Rarely, if ever, is the entire “population” studied. From study of the “sample,” we may extrapolate to the “population.” This extrapolation has a level of uncertainty that is estimated statistically. Use the terms “sample,” “cohort,” or “group” instead of “population” when referring to the actual animals studied.

### 3.35 Present/Presented

“Present,” as a verb, means to come forward as a patient. In veterinary medicine, patients do not “present” on their own but are “presented” by their owners for evaluation. Thus, “the dog was presented for evaluation of diarrhea” and not “the dog presented with diarrhea” or (worse) “the dog presents diarrhea.”

### 3.36 Predominant/Predominate

“Predominate” is a verb meaning to be the strongest or main element or to be largest in number or amount; “predominant” is an adjective; “predominantly” is an adverb.

### 3.37 Principal/Principle

“Principle” is a noun meaning “a rule of action or conduct”; “principal” usually is an adjective meaning “most important.” Rarely, “principal” is a noun meaning the head administrator of a school or one of the main participants in a business transaction.

### 3.38 Receiver operating characteristic curve/Receiver operator characteristic curve

“Receiver operating characteristic curve” is the correct term; not “receiver operator characteristic curve.”

### 3.39 Reference range/Normal range

“Reference range” is preferred to “normal range” to avoid the impression that the values used to determine the “range” followed a normal (Gaussian) distribution or that the animals with values within that range are necessarily normal. The boundaries of the “reference range” are referred to as the “reference limits.”

### 3.40 Significant/Substantial

To avoid confusion, the Journal's policy is to use the word “significant” only when referring to information that has been evaluated statistically. Substitute “substantial” or “clinically relevant” if the information was not evaluated statistically. For example, “the dog's serum creatinine concentration was substantially higher on hospital Day 2” (unless you used a statistical test to compare the concentration on Day 2 to the concentration on Day 1). Also, according to Journal policy, “statistically significant” is considered redundant; saying the “results were significantly different” suffices.

### 3.41 That/Which

“That” introduces a *restrictive* clause; “which” introduces a *nonrestrictive* clause. Thus, it is correct to say that “dogs, which have 4 legs, make wonderful pets” and also to say, “dogs that have 3 legs have a difficult time getting around.”

### 3.42 Toxicity/Toxicosis/Intoxication

Toxicity is a property of the compound, and not a clinical syndrome. *Toxic* agents *intoxicate* animals causing characteristic clinical signs and disease (*toxicosis* or *toxicoses*). Toxic agents cause adverse effects as a result of their toxicity. Thus, antineoplastic agents are often toxic and as a result of their toxicity cause intoxication resulting in adverse effects, including hematologic or gastrointestinal toxicoses. Note that a toxin is always of biological origin, whereas toxic substances (poisons) can be of any origin.

### 3.43 Treatment/Therapy

“Treatment” typically indicates intent to cure a patient of a disease whereas “therapy” indicates intent to rehabilitate. The Journal's editorial style is to use “treatment” in most instances but, because of established usage and convention, it continues to accept the terms “radiation therapy,” “chemotherapy,” and “fluid therapy.”

### 3.44 Ultrasonography/Ultrasound

“Ultrasound” refers to the vibrations used to perform the procedure of “ultrasonography.” Do not use “ultrasound” as a noun to refer to the procedure. “Ultrasound” also should not be used as a verb.

### 3.45 Unremarkable

Do not use the word “unremarkable” to describe the situation in which results of an examination did not detect abnormalities. The fact that you are referring to them (ie, remarking on them) makes them remarkable. Use the expression “abnormalities were not detected on examination.”

### 3.46 Varying/Various/Variable

“Varying” means exhibiting or undergoing change. “Various” or “variable” means of different kinds. For example, “lesions of various sizes” or “lesions of variable size,” but not “lesions of varying size” (unless the lesions were changing size right before your eyes!).

### 3.47 Whereas/While

Instead of using “while” as a subordinating conjunction, use “whereas.” “While” has the connotation of time and thus can be misinterpreted.

